# Trop-2 expression in non–small cell lung cancer

**DOI:** 10.1371/journal.pone.0321555

**Published:** 2025-04-15

**Authors:** Peiwen Kuo, Emon Elboudwarej, Marianna Zavodovskaya, Kai-Wen Lin, Chingwei V. Lee, Lauri Diehl, Jilpa Patel, Sabeen Mekan, Juliane M. Jürgensmeier

**Affiliations:** 1 Gilead Sciences, Inc., Foster City, California, United States of America; 2 Gilead Sciences, Inc., Morris Plains, New Jersey, United States of America; Kameda Medical Center, JAPAN

## Abstract

Trophoblast cell-surface antigen 2 (Trop-2) is highly expressed in non–small cell lung cancer (NSCLC) and has become an attractive target for antibody-drug conjugates (ADCs). ADC tumor target expression is essential in investigating the predictive value of Trop-2 and Trop-2 ADC efficacy. Although Trop-2 mRNA expression in NSCLC has been described, protein-level expression is poorly understood. We investigated Trop-2 expression landscape across multiple data and sample sets to characterize mRNA expression and address the gap in protein-expression profiling. Trop-2 expression was analyzed using available mRNA, mutation, and protein data in three datasets: (1) The Cancer Genome Atlas (TCGA) included clinical-pathological and survival data in NSCLC adenocarcinoma and squamous cell carcinoma; (2) sample set 1 (adenocarcinoma) and (3) sample set 2 (adenocarcinoma, squamous cell carcinoma) underwent sequencing and immunohistochemistry for Trop-2 RNA, protein (Robust Prototype Assay, SP295 clone) and mutation analysis. Trop-2 was highly expressed in NSCLC and expression was similar in adenocarcinoma and squamous cell carcinoma and across baseline characteristics including patient age, sex, and tumor stage. Trop-2 expression was not associated with clinically relevant genetic alterations. Trop-2 was not a prognostic factor in NSCLC (TCGA survival data). High Trop-2 expression in NSCLC was independent of evaluated baseline characteristics, histology, and driver alterations. Trop-2 protein expression at any level was observed in 82% to 90% of NSCLC across sample sets; similar proportions of adenocarcinoma and squamous cell carcinoma expressed Trop-2. These data support broad Trop-2 ADC use in NSCLC.

## Introduction

Lung cancer is the leading cause of cancer deaths worldwide with more than 200,000 new cases and over 100,000 deaths annually [1]. Non–small cell lung cancer (NSCLC) comprises 85% of all lung cancers, and adenocarcinoma and squamous cell carcinoma are the most frequent histological subtypes, while the not otherwise specified (NOS) subtype composed of heterogeneous tumors are relatively less frequent [2–4]. Currently, two-thirds of NSCLC is diagnosed as advanced or metastatic disease, and platinum-based chemotherapy and immune checkpoint inhibitors in combination or given sequentially are the standard of care for patients without actionable genomic alterations. Molecularly targeted therapies are approved for specific oncogene-driven disease including genetically altered *EGFR, ALK*, and others. Although immunotherapy and small molecule tyrosine kinase inhibitors have resulted in substantial survival benefits, survival rates and overall cure in NSCLC remain low; the 5-year survival rate of advanced NSCLC is less than 5% [5].

Antibody-drug conjugates (ADCs) are a promising therapeutic strategy, with certain ADCs intentionally designed to deliver potent activity to both trophoblast cell-surface antigen 2 (Trop-2)–expressing cancer cells and to neighboring cancer cells through a bystander effect [6]. Trop-2, also known as tumor-associated calcium signal transducer 2 (TACSTD2), is highly expressed in multiple solid tumors and has emerged as an attractive ADC target [7]. Sacituzumab govitecan-hziy is a first-in-class ADC composed of a Trop-2–directed humanized monoclonal antibody (hRS7 IgG1ĸ) coupled to SN-38, an active metabolite of the topoisomerase I inhibitor irinotecan and is being evaluated in NSCLC (NCT05089734, NCT05186974, NCT05609968, NCT05633667, NCT03337698). As the target protein expression of ADCs drives localized delivery of cytotoxic payloads, characterization of Trop-2 expression in NSCLC is essential to our exploration of the relationship between Trop-2 ADC efficacy and tumor Trop-2 expression. Trop-2 mRNA expression in NSCLC has been described in the literature using sample cohorts of varying sizes [8,9]. However, protein-expression analysis and data interpretation have been limited due to frequent use of antibodies of unknown or poor specificity. Here, we examined Trop-2 expression in NSCLC using available mRNA and protein data in three independent datasets. Our data show high expression of Trop-2 mRNA and protein expression in NSCLC independent of baseline characteristics, histology, and molecular alterations of interest in three independent datasets.

## Materials and methods

### Datasets

Trop-2 prevalence was analyzed in three independent datasets: (1) The Cancer Genome Atlas (TCGA) data, (2) sample set 1, and (3) sample set 2, described in detail below. TCGA NSCLC adenocarcinoma cohort (LUAD) included 566 patients in which a subset included whole transcriptome (RNAseq, *n* =  510), whole-exome sequencing (WES; *n* =  566), and 5-year overall survival with Trop-2 mRNA data (*n* =  508) [10]. The NSCLC squamous cell carcinoma (LUSC) cohort included 487 patients with RNAseq (*n* =  484), WES (*n* =  484), and survival data with Trop-2 mRNA data (*n* =  481). TCGA tumor specimens selected for study were high in tumor nuclei content. RNAseq data were downloaded from UCSC Xena (http://xena.ucsc.edu, RRID:SCR_018938) from the University of California, Santa Cruz. WES mutation data were downloaded from cBioportal (http://www.cbioportal.org, RRID:SCR_014555). Clinico-pathologic characteristics in TCGA including sex, race, age, histological subtype, stage and molecular features of the LUAD and LUSC datasets were previously described [10,11]. Data from TCGA was accessed on August 14^th^ 2020, using version 2016-09-01.

Sample set 1 included procured NSCLC adenocarcinoma formalin-fixed paraffin-embedded (FFPE) tumor specimens from the US (*n* =  107). Tumor specimens were selected based on several parameters, including high tumor content and low necrosis criteria based on pathologist review of hematoxylin and eosin (H&E) staining. Samples were also selected with associated mutation data derived from a tumor mutation panel (TruSight, Illumina, San Diego, CA). The sample set contained ~ 25% of samples with *TP53* mutations as well as ~ 25% with *KRAS* mutations. Samples with other driver mutations were not represented in the sample set. Therefore, sample set 1 was not intended to reflect the prevalence of mutations occurring in NSCLC. RNAseq conducted for sample set 1 is described in the below sequencing section. In total, this dataset includes transcriptomic (*n* =  103) and Trop-2 immunohistochemistry (*n* =  107) and tumor panel mutation data (*n* =  110). Sample set 2 included adenocarcinoma (*n* =  102) and squamous cell carcinoma (*n* =  56) collected by surgical resections, excisions, or biopsy procedures. Specimens were primarily from the US with few from outside the US (Europe and Australia). Data for Trop-2 immunohistochemistry (*n* =  158), RNAseq (*n* =  48), and WES (*n* =  46) were included in our analyses. RNA sequencing methodology is also described below. Overall, with regard to demographics, specimens from sample sets 1 and 2 were relatively balanced for males versus females and ages < vs ≥  65, however, weight was not available. Furthermore, tissues from both sample sets were collected from multiple institutions and the preparation of FFPE tissues were conducted by each site/institution’s standard operating procedures. Tissues ranged from 1 to 27 years old. For sample set 1, tissue specimens were acquired from Discovery Life Sciences and were obtained from approved IRB protocols including an IRB approved waiver of consent. De-identified samples were accessed 29/06/2019 and the study was conducted from 2020 to 2022. Specimen collection for sample set 2 was conducted under Western Institutional Review Board-Copernicus Group (WCG IRB)-approved protocol (20190845) with signed informed consent obtained for all specimens. For sample set 2, all samples were accessed 07/10/2019–07/06/2021.

### Immunohistochemistry

FFPE tumor specimens were evaluated by Trop-2 immunohistochemistry to characterize the amount of protein expressed using the anti–Trop-2 SP295 clone (Spring Bioscience Co., Pleasanton, CA) at Roche Tissue Diagnostics (Tucson, AZ) using the SP295 Robust Prototype Assay (RPA). Trop-2 protein membrane histological scores (H-scores) on a scale of 0–300 were calculated using the formula: H-score =  [(I1 ×  P1) +  (I2 ×  P2) +  (I3 ×  P3)], capturing both intensity (I1, I2, I3) and proportion of Trop-2 membrane-positive tumor cells at each corresponding intensity (P1, P2, P3, respectively). The proportion of Trop-2 membrane-positive tumor cells was also scored at a low magnification (4 × objective; % membrane-positive tumor cells). Scoring of both Trop-2 metrics was conducted by a pathologist (Roche Tissue Diagnostics, Tucson, AZ). The Aperio ImageScope (RRID:SCR_020993) was used for image capture at 20 × objective magnification of anti–Trop-2, IgG negative control, and H&E-stained specimens. PD-L1 immunohistochemistry assay was conducted to analyze protein expression using the clone 22C3 and Tumor Proportion Score (TPS) scoring in sample set 1 (Mosaic Laboratories, Lake Forest, CA). There was insufficient tissue for programmed death-ligand 1 (PD-L1) immunohistochemistry analysis in sample set 2.

### RNAseq in sample set 1 and sample set 2

In sample set 1, dual nucleic acid extraction from FFPE tumor specimens (Allprep, Qiagen) were conducted. RNA exome-capture protocol was used (TruSeq RNA Exome kit, Illumina) and libraries were sequenced to a depth of 100M pair-end reads (200M total) (Q2 Lab Solutions, Morrisville, NC). In sample set 2, dual extraction of nucleic acids from FFPE samples was conducted using the MagMax FFPE kit (Thermo Fisher Scientific, Waltham, MA). RNA library preparation was performed using the TruSeq RNA Exome kit (Illumina). Libraries were sequenced to a depth of 100M paired-end reads (200M total) (Fulgent Genetics, Temple City, CA). WES library preparation was performed using IDT xGen WES Library Prep Kit (Integrated DNA Technologies, Redwood City, CA) and sequenced to a depth of 150 × mean target coverage. In both specimen sets, macrodissection was employed in instances when tumor content was less than 50% to enrich for tumor content for nucleic acid extraction. RNAseq quality control revealed high-quality sequencing data based on percentage of unique mapped reads in exonic regions. RNA data processing of FASTQ was done using nf-core/rnaseq pipeline (version 3.10.1) [12]. RNAseq data was utilized to characterize RNA expression levels, which were expressed as transcripts per million (TPM) reads in the log2 (TPM + 0.01) format. TPM values were transcript counts normalized to gene length and then sequencing depth to convey relative measure of expression, enabling accurate cross-sample comparisons.

### Trop-2 clone specificity

Five Trop-2 antibody clones, including hRS7 IgG1ĸ of sacituzumab govitecan, rabbit monoclonal SP295 (Abcam Cat# ab227691, RRID:AB_3075505), mouse monoclonal ENZ-ABS380 (Enzo Life Sciences Cat# ENZ-ABS380), rabbit monoclonal R001 (Sino Biological Cat# 10428-R001), and goat polyclonal AF650 (R&D systems Cat# AF650, RRID:AB_2205667) were assessed for their binding specificity to Trop-2 using ELISA. Maxisorp immuno 96-well plates (Thermo Fisher) were coated with 1ug/ml Trop-2 or EPCAM (Sino Biological Cat# 10694-H08H) in PBS. Serial dilutions of anti–Trop-2 antibodies were added to antigen coated plates for 1-hour binding at room temperature. Unbound antibodies were washed off using wash buffer (PBS and 0.05% Tween20). Bound anti–Trop-2 antibodies were detected by horseradish peroxidase-linked goat anti-human (Thermo Fisher Cat# 31430, RRID:AB_228307) or anti-rabbit Fc secondary antibody (Jackson Laboratories cat# 111-035-008, RRID:AB_2337937) and developed using chromogenic substrate (3, 3’, 5, 5’-tetramethylbenzidine, TMB) solution per manufacturer’s instructions. Plates were read at absorbance OD450 nm using a SpectraMax M5e (Molecular Devices, LLC).

### Statistical analyses

Wilcoxon rank-sum and Kruskal–Wallis tests were used for continuous variables, to assess differences between two or multiple groups, respectively. The Spearman correlation coefficient was used for associations between continuous variables. Kaplan–Meier curves were generated and Cox proportional hazards models were used to assess the association between Trop-2 mRNA expression and survival outcomes (R version 4.0.5, RRID:SCR_001905 and GraphPad Prism 8.1.2, RRID:SCR_002798, La Jolla, CA). Both unadjusted and adjusted multivariate Cox models were employed to account for the potential confounding effects of various disease metrics; a fully adjusted model included as covariates: median age, microsatellite instability (MSI) score, sex, and median mutation count as a measure of mutational burden, as well as key NSCLC mutations of interest (*ALK, ROS1*, *RET*, *KRAS*, and *EGFR*).

## Results

### Characterization of Trop-2 mRNA expression and prognostic value in NSCLC in TCGA dataset

**Fig 1:**
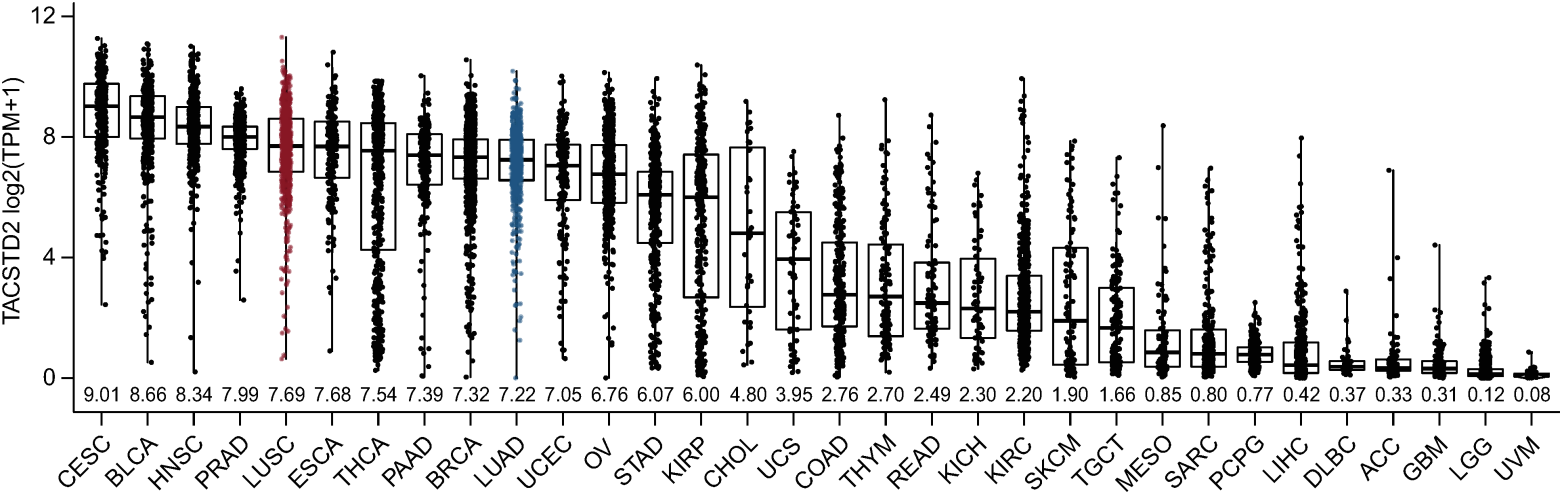
Trop-2 mRNA expression distribution across multiple solid tumor types in TCGA. Trop-2 expression in NSCLC LUAD and LUSC is highlighted in blue. Trop-2 mRNA expression is shown as log2 of the transcript count per million (log2TPM+1) and median expression is shown along x-axis for each indication. Abbreviations: ACC, adrenocortical carcinoma; BLCA, bladder urothelial carcinoma; BRCA, breast invasive carcinoma; CESC, cervical squamous cell carcinoma and endocervical adenocarcinoma; CHOL, cholangiocarcinoma; COAD, colon adenocarcinoma; DLBC, lymphoid neoplasm diffuse large B-cell lymphoma; ESCA, esophageal carcinoma; GBM, glioblastoma; HNSC, head and neck squamous cell carcinoma; KICH, kidney chromophobe; KIRC, kidney renal clear cell carcinoma; KIRP, kidney renal papillary cell carcinoma; LGG, low grade glioma; LIHC, liver hepatocellular carcinoma; MESO, mesothelioma; OV, ovarian serous cystadenocarcinoma; PAAD, pancreatic adenocarcinoma; PCPG, pheochromocytoma and paraganglioma; PRAD, prostate adenocarcinoma; READ, rectum adenocarcinoma; SARC, sarcoma; SKCM, skin cutaneous carcinoma; STAD, stomach adenocarcinoma; TACSTD2, tumor-associated calcium signal transducer 2; TGCT, tenosynovial giant cell tumor; THCA, thyroid carcinoma; THYM, thymoma; UCEC, uterine corpus endometrial carcinoma; UCS, uterine carcinosarcoma; UVM, uveal melanoma.

**Fig 2:**
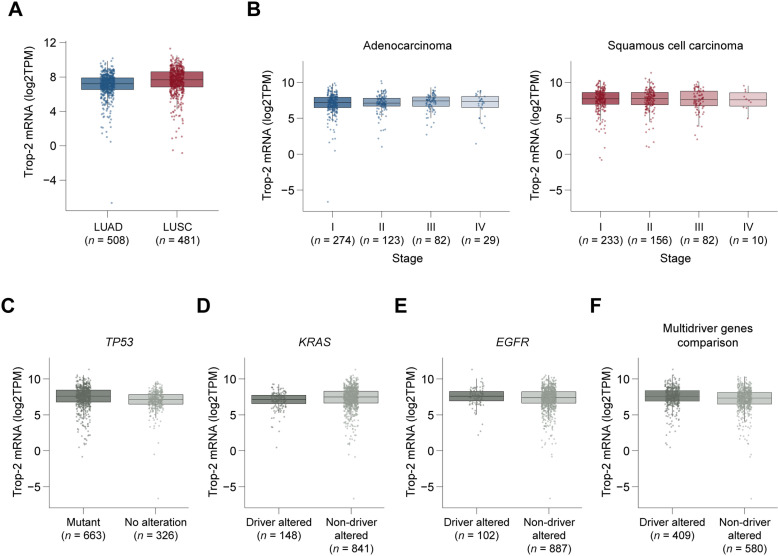
Trop-2 mRNA expression in NSCLC in TCGA datasets. Comparison of Trop-2 mRNA expression is shown in LUAD and LUSC histological subtypes (A), across stages I-IV for each subtype (B), mutation status of LUAD and LUSC combined of *TP53* (C), *KRAS* (D), *EGFR* (**E**) and grouping of multiple oncogenes including *EGFR*, *MET*, *ERBB2*, *BRAF*, *NTRK*, *PI3KCA*, *ALK*, *ROS*, *RET*, but excluding *KRAS* (F). Abbreviations: ALK, anaplastic lymphoma kinase; BRAF, v-raf murine sarcoma viral oncogene homolog B1; EGFR, epidermal growth factor receptor; ERBB2, human epidermal growth factor receptor 2; KRAS, Kirsten rat sarcoma viral oncogene homolog; MET, mesenchymal epithelial transition factor; NTRK, neurotrophic tyrosine receptor kinase; PIK3CA, phosphatidylinositol 4,5 bisphosphate 3 kinase catalytic subunit alpha; RET, ret proto oncogene; ROS, c ros oncogene 1; TP53, tumor protein 53.

We analyzed comprehensive genomic data across numerous solid tumor types from TCGA to examine Trop-2 mRNA expression and determine the relative expression of Trop-2 in NSCLC in a pan-cancer comparison of 32 solid tumor indications. NSCLC LUAD and LUSC are among the top one-third of indications ranked by Trop-2 gene expression (Fig 1). Various solid tumors are quite low in Trop-2 mRNA expression based on TCGA data, including uveal melanoma and glioblastoma. In NSCLC, Trop-2 gene expression was similar in adenocarcinoma (LUAD) and squamous cell carcinoma (LUSC) and expressed at similar levels between histological subtypes and across stages I through IV (Fig 2A and 2B). Additionally, Trop-2 expression was independent of sex, race, and age (S1 Fig).

**Fig 3:**
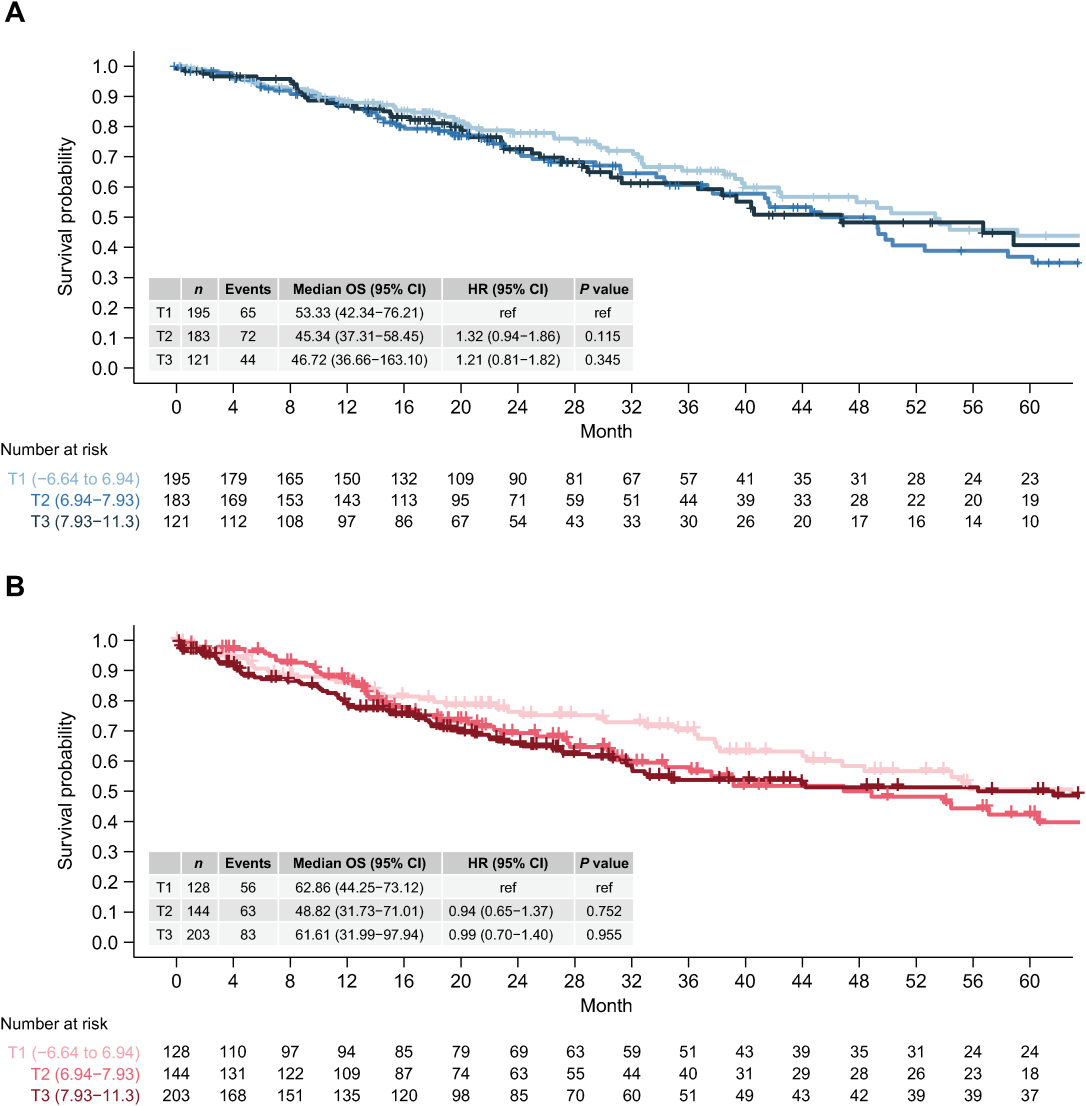
Prognostic analysis of Trop-2 mRNA expression in NSCLC in TCGA. Kaplan–Meier analysis of overall survival in LUAD (**A**) and LUSC (**B**) patients based on Trop-2 mRNA expression split into tertiles with upper, middle, and lower thirds indicated as light, medium, and dark colors, respectively, is shown. Patient numbers and events are summarized in 4-month increments. The table summarizes each Trop-2 tertile median OS and 95% CI and HR with 95% CI based on multivariate Cox proportional hazards model. Results were adjusted for age, MSI score, sex mutational load, and specific gene alterations including *ALK*, *ROS1*, *RET*, *EGFR*, and *KRAS*. Abbreviations: OS, overall survival; ref, reference.

A subset of NSCLC, in particular adenocarcinoma, harbor tumor suppressor and driver alterations with clinical relevance. We therefore compared Trop-2 expression of samples with wildtype and altered genes, including *TP53*, *KRAS*, and *EGFR*. Trop-2 mRNA expression was similar in the presence or absence of driver alterations (Fig 2C–2E). When we combined all cases with driver alterations *EGFR*, *MET*, *ERBB2*, *BRAF*, *NTRK*, *PI3KCA*, *ALK*, *ROS1*, and *RET* (but excluding *KRAS*, which are more frequent and therefore analyzed separately), into a single group, we also did not observe a difference in Trop-2 gene expression compared with samples without these alterations (Fig 2F). We also examined the relationship between Trop-2 and PD-L1 mRNA expression, in which there was no correlation in either adenocarcinoma or squamous cell carcinoma (S2 Fig). Altogether, Trop-2 expression did not vary across baseline characteristics and tumor molecular features of interest in NSCLC. Unadjusted Cox proportional hazards models showed that Trop-2 was not a prognostic factor for 5-year overall survival in TCGA LUAD [T2 versus T1 HR (95% confidence interval [CI]) =  1.32 (0.94–1.86) *P* >  0.05; T3 versus T1 HR (95% CI) =  1.21 (0.81–1.82) *P* >  0.05] or LUSC [T2 versus T1 HR (95% CI) =  0.94 (0.65–1.37) *P* >  0.05; T3 versus T1 HR (95% CI) =  0.99 (0.70–1.39) *P* >  0.05] (Fig 3). Multivariable Cox proportional hazards models confirmed that Trop-2 was not associated with overall survival in both LUAD and LUSC, even after adjusting for potential confounders (S3 Fig).

### Trop-2 expression in an NSCLC adenocarcinoma sample set 1

**Fig 4:**
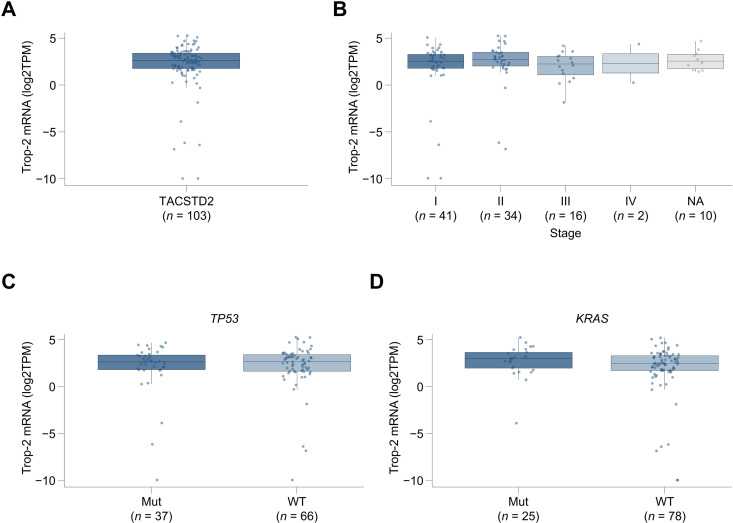
Trop-2 mRNA expression in an NSCLC adenocarcinoma sample set 1. Trop-2 mRNA expression distribution in the sample set (A), comparison of expression across stages I through IV (B), between mutant and wildtype *TP53* (**C**) and *KRAS* (**D**) is shown. NA indicates samples without stage information. Abbreviations: Mut, mutant; WT, wildtype.

**Fig 5:**
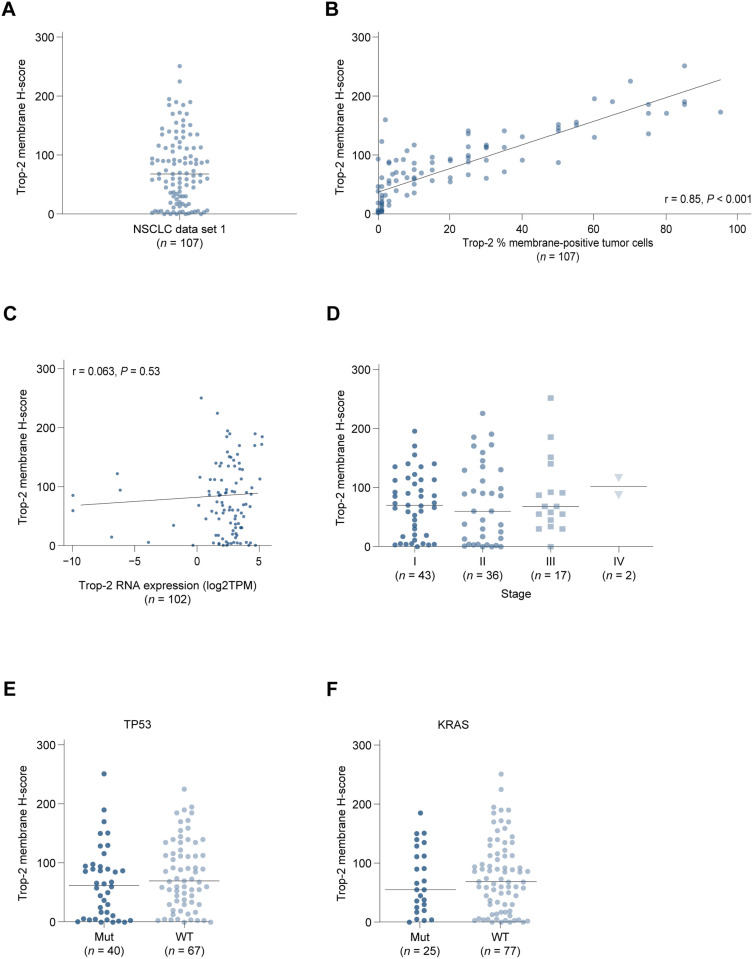
Trop-2 protein expression in an NSCLC adenocarcinoma sample set 1. Trop-2 protein expression is shown as membrane H-score (A). The correlation between Trop-2 membrane H-score and the percentage of Trop-2-membrane positive tumor cells (at 4 × objective magnification) is shown in the scatter plot (r =  0.85, ****P**** <  0.001) (B). Correlation between Trop-2 mRNA and protein expression is shown in a scatter plot (C). Trop-2 protein expression across stages I through IV (D), in mutant and wildtype *TP53* (E), and *KRAS* (**F**) is shown.

We utilized RNAseq and mutation and protein immunohistochemistry data in sample set 1 to explore Trop-2 expression in adenocarcinoma. Trop-2 mRNA expression was similar across stages I through IV (Fig 4A and 4B) and was similar in samples with mutant and wildtype *TP53* and *KRAS* (Fig 4C and 4D). Similarly, Trop-2 gene expression was also not associated with sex, race, or age (S4 Fig). These observations were consistent with our analyses of TCGA data above. Sacituzumab govitecan targets membrane expression of Trop-2 and we therefore characterized protein expression on the tumor cell surface. Trop-2 protein was highly expressed in the adenocarcinoma sample set with a median H-score of 68 and range of 0–251. 95% and 82% of specimens were found to express any level of Trop-2 (greater than zero), based on H-score and percentage of membrane-positive tumor cells metrics, respectively. In addition, Trop-2 expression scores as membrane H-score and percentage of membrane-positive tumor cells highly correlated (r =  0.85, *P* <  0.001) (Fig 5A and 5B). In regard to the relationship between Trop-2 mRNA and protein expression, we observed limited correlation (Fig 5C). Our examination of Trop-2 protein expression association with baseline characteristics revealed similar levels of expression across tumor stage and *TP53* and *KRAS* mutation status (Fig 5D–5F). Trop-2 and PD-L1 protein expression also did not correlate (S5 Fig). Trop-2 expression was not associated with sex or age, therefore, protein-level findings were consistent with the mRNA data (S6 Fig). Overall, Trop-2 mRNA and protein expression did not vary across evaluated baseline characteristics and alterations of interest and Trop-2 mRNA expression observations in sample set 1 were consistent with our analysis of the TCGA dataset.

### Exploration of Trop-2 expression in sample set 2

**Fig 6:**
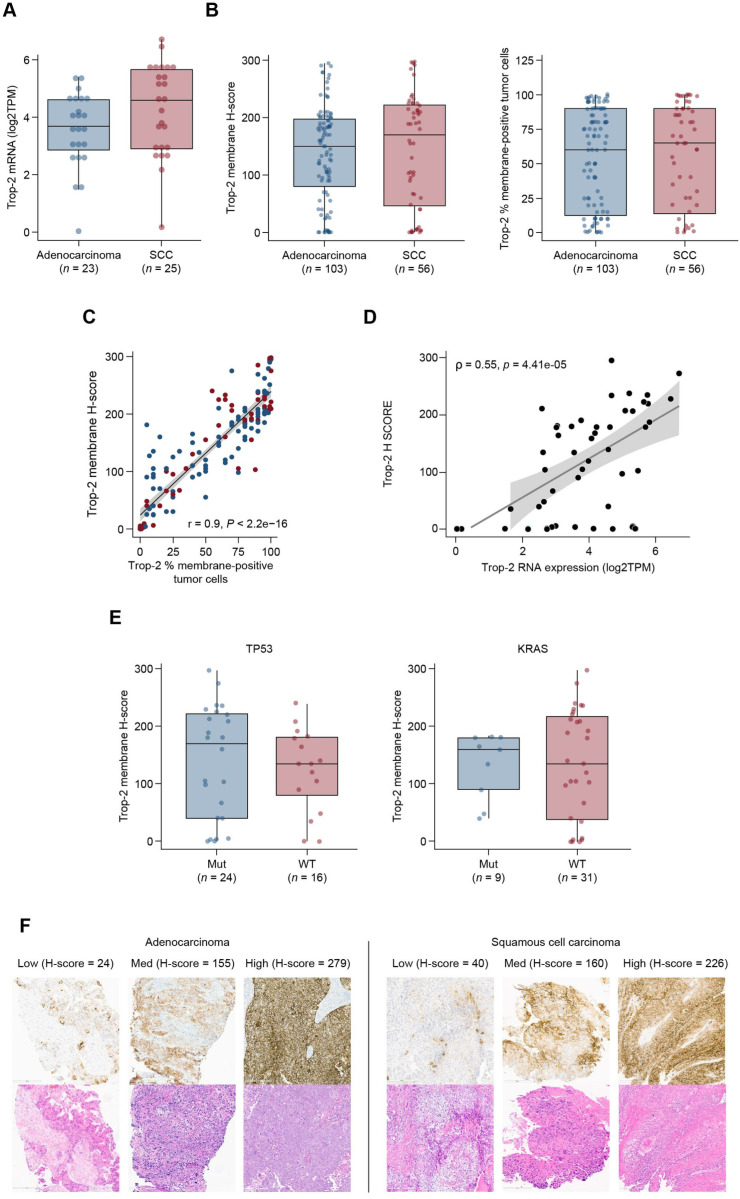
Trop-2 mRNA and protein expression in an NSCLC adenocarcinoma and squamous cell carcinoma sample set 2. Trop-2 mRNA and protein expression distribution across histological subtypes is shown (**A**, **B**). Trop-2 H score and % membrane positive tumor cells correlation depicted in scatter plot (C). Correlation between Trop-2 mRNA and protein expression is shown in a scatter plot (D). Trop-2 protein expression relative to TP53- and KRAS-mutated and WT specimens is shown (E). NSCLC Trop-2 protein immunohistochemistry in NSCLC adenocarcinoma and squamous cell carcinoma sample set 2. Representative images of Trop-2 protein expression in adenocarcinoma and squamous cell carcinoma is shown (F). Trop-2 expression in adenocarcinoma specimens at low (H-score 24), medium (H-score 155) and high (H-score 297) levels are illustrated (left images). Trop-2 expression in squamous cell carcinoma specimens at low (H-score 40), medium (H-score 160) and high (H-score 226) levels are shown (right images). Corresponding H&E images are shown below immunohistochemistry images. All images were captured at 20 × objective magnification. Abbreviations: H&E, hematoxylin and eosin; SCC, squamous cell carcinoma.

To validate observations from datasets described previously, we examined Trop-2 mRNA and protein expression in another independent sample set (sample set 2). With the available data, we observed that Trop-2 mRNA and protein expression were similar in adenocarcinoma and squamous cell carcinoma, which was consistent with both TCGA dataset and sample set 1 observations. Both histological subtypes had a similar proportion of specimens expressing Trop-2 defined as an H-score >  0 (92% in adenocarcinoma and 91% in squamous cell carcinoma) (Fig 6A and 6B). Trop-2 expression as membrane H-score and percentage of positive tumor cells also highly correlated in this dataset, consistent with sample set 1 (Fig 6C). In line with previous observations that Trop-2 is highly expressed in NSCLC, 90% of tissue specimens in this sample set expressed Trop-2 at any level ( > 0; using either the percentage of Trop-2 positive tumor cells or membranous H-scores). The H-score medians were 150 and 170 for adenocarcinoma and squamous cell carcinoma, respectively, and the H-score range was 0–298. In regard to the relationship between Trop-2 mRNA versus protein expression, we observed a modest correlation (Fig 6D). As evaluated in previous data and sample sets, we found that Trop-2 expression was independent of *TP53* and *KRAS* mutation status (Fig 6E). Trop-2 expression was also similar between sexes and did not correlate with age (S7A and S7B Fig). For a subset of the tissue specimens, tissue classification as primary or metastatic was available, and comparison of Trop-2 expression was found to be similar in these tissues (S7C Fig). Observations in adenocarcinoma from sample set 2 were similar to sample set 1, which was composed of only adenocarcinoma specimens. Across tumor specimens, a range of Trop-2 staining was observed with representative images depicting low, medium, and high expression (based on H-scores) in adenocarcinoma and squamous cell carcinoma NSCLC (Fig 6F).

## Discussion

Examination of Trop-2 as the target for ADCs is important to assess the prognostic and predictive value of Trop-2 expression in clinical trials with Trop-2–directed ADCs. We therefore investigated Trop-2 expression across three independent datasets to describe Trop-2 expression and its relationship to baseline characteristics and molecular features of interest in NSCLC. Here, we report that Trop-2 mRNA and protein expression were similar across all demographic features examined with regard to patient and tumor baseline characteristics, including patient age, sex, and race, as well as NSCLC tumor histological subtype (adenocarcinoma and squamous cell carcinoma), stage, and clinically relevant driver alterations. Analysis of the relationship between Trop-2 mRNA versus protein expression revealed varying degrees of correlation in the two sample sets (minimal in sample set 1 and modest in sample set 2). This can be explained by Trop-2 mRNA data, which were from the whole tumor, whereas Trop-2 protein analysis was specific to tumor membrane expression. Trop-2 mRNA expression also did not correlate with PD-L1, which may suggest the potential activity of SG across all NSCLC patients regardless of PD-L1 expression levels. Furthermore, Trop-2 was not a prognostic factor in NSCLC adenocarcinoma or squamous cell carcinoma based on RNA expression data derived from TCGA data. The similar distribution in Trop-2 expression we observed supports the concept that the broad NSCLC patient population is amenable to Trop-2 ADC therapy based on Trop-2 expression.

The characterization of Trop-2 protein expression as it relates to patient and tumor baseline characteristics has been inconsistent in the literature. Several groups have reported significant associations between Trop-2 and tumor grade, stage, lymph node metastasis in adenocarcinoma or squamous histology, although correlations were not consistently observed across histological subtypes [8,13–15]. Conversely, others have also reported a lack of association between Trop-2 and baseline characteristics [16]. One limitation to the interpretation of the multiple publications reporting significant associations between Trop-2 protein expression measured by immunohistochemistry and demographics is the use of antibodies with unknown specificity for Trop-2. We interrogated the specificity of several anti-Trop-2 antibodies in the literature and identified those that cross-reacted with EPCAM, a finding that suggests limited interpretation of certain protein expression data. In contrast, the SP295 clone, with confirmed specificity for Trop-2, was developed as an RPA at Ventana, Roche-Tissue Diagnostics (S8 Fig). Omori and colleagues, who also utilized the SP295 clone in their immunohistochemistry assay, reported high frequency of Trop-2 positive cases in their NSCLC cohort (*n* =  130) with at least 88% considered positive, defined as intensities I2 and I3. Trop-2 was also not associated with age, sex, smoking status, stage, and *EGFR* alteration, in their NSCLC cohort [16]. Observations between our studies, which utilize the SP295 clone, were consistent; however, the authors used a different scoring metric than we have used in our analyses. In addition, the assay optimization Omori et al. used may result in a different staining intensity compared with the assay used in our study (at Roche Tissue Diagnostics); therefore, data may not be directly comparable, while consistent in the conclusion.

The prognostic value of Trop-2 in NSCLC also remains inconsistent based on literature. Trop-2 was initially identified as a transmembrane glycoprotein highly expressed in trophoblast cells, which possess stem-like and proliferative properties akin to cancer cells [17]. Cell line–based analyses have shown Trop-2 modulation of distinct signaling pathways (e.g., MAPK, CREB, PI3K/AKT, pSTAT1/3, NFKB, and RB) involved in tumorigenesis through enhanced proliferation, migration, and epithelial-to-mesenchymal transition [18–20]. Conversely, Trop-2 has been shown to abrogate NSCLC tumor cell growth through binding to and blocking IGF-1 signaling [21]. Therefore, data exist to support the hypotheses that Trop-2 could be either a negative, positive, or not a prognostic factor if it serves tumor promoting and abrogating functions. Trop-2 was not associated with OS when considering low/no Trop-2 (I0, I1) versus moderate/high (I2, I3) protein expression and this result was based on the Trop-2-specific SP295 clone [16]. Trop-2 has been described as a positive prognostic factor in NSCLC adenocarcinoma (overall and progression-free survival) [15]. Conversely, several studies report the opposite observation with Trop-2 expression associated with worse survival [8,13,22]. Additional analysis using the appropriate immunohistochemistry assay will provide supporting data if Trop-2 is not a prognostic factor in NSCLC.

In summary, our data show that Trop-2 is highly expressed in NSCLC independent of histological subtypes, other baseline characteristics or driver alterations. In two tumor sample sets, Trop-2 expression at any level ( > 0%) was observed in 82% and 90% (sample set 1 and 2, respectively) based on the SP295 RPA. These data support clinical trials with Trop-2 ADCs in NSCLC. However, the elucidation of Trop-2 as a prognostic or predictive biomarker will require additional analyses in a large clinical trial setting and exploration of potential treatment-induced changes in Trop-2 expression.

## Supporting information

S1 Fig Trop-2 expression across baseline characteristics in NSCLC in TCGA. Trop-2 expression by histological subtype and across sex, race, and age is shown. Abbreviations: NSCLC, non–small cell lung cancer; TCGA, The Cancer Genome Atlas; TPM, transcript count per million; Trop-2, trophoblast cell surface antigen 2.
(TIF)

S2 Fig Trop-2 and PD-L1 (CD274) mRNA expression correlation in adenocarcinoma (LUAD) and squamous cell carcinoma (LUSC) is shown. Abbreviations: CD, cluster of differentiation; LUAD, lung adenocarcinoma; LUSC, lung squamous cell carcinoma; PD-L1, programmed death-ligand 1.
(TIF)

S3 Fig Multivariate Cox proportional hazards analysis of Trop-2 and other covariates including baseline demographics and tumor characteristics in NSCLC adenocarcinoma (A) and squamous cell carcinoma (B) in TCGA. Abbreviations: ALK, anaplastic lymphoma kinase; EGFR, epidermal growth factor receptor; KRAS, Kirsten rat sarcoma viral oncogene homolog; Mut, mutant; RET, ret proto oncogene; ROS1, c ros oncogene 1; TACSTD2, tumor-associated calcium signal transducer 2; WT, wildtype.
(TIF)

S4 Fig Trop-2 mRNA expression across baseline characteristics in sample set 1. Trop-2 mRNA expression across sex (A), race (B) and correlation with age (C) are shown.
(TIF)

S5 Fig Correlation between Trop-2 and PD-L1 protein expression is shown. (TIF)

S6 Fig Trop-2 protein expression across baseline characteristics in sample set 1. Trop-2 expression as H-score across sex (A) and correlation with age (B) is shown.
(TIF)

S7 Fig Trop-2 protein expression across baseline characteristics in sample set 2. Trop-2 protein expression as H-score across sex (A), correlation with age (B), and between primary and metastatic tumors (C) is shown.
(TIF)

S8 Fig Examination of multiple anti–Trop-2 antibodies for binding to Trop-2 and EPCAM (Trop-1) by ELISA. Humanized monoclonal antibody hRS7 IgG1ĸ of sacituzumab govitecan (A), rabbit monoclonal anti–Trop-2 (clone SP295) (B), mouse monoclonal anti–Trop-2 (clone ENZ-ABS380) (C), and rabbit monoclonal anti–Trop-2 (clone 001) specifically bind Trop-2 (D), and goat anti–Trop-2 (clone AF650) cross-reactivity to EPCAM/Trop-1 are shown (E). Abbreviations: BSA, bovine serum albumin; EPCAM, epithelial cell adhesion molecule; OD, optical density.
(TIF)
