# Supplementary figures and images for: Trop-2 expression in non–small cell lung cancer
Source: PLoS One. 2025 Apr 15;20(4):e0321555. doi: 10.1371/journal.pone.0321555 (PMC11999141; doi:10.1371/journal.pone.0321555)

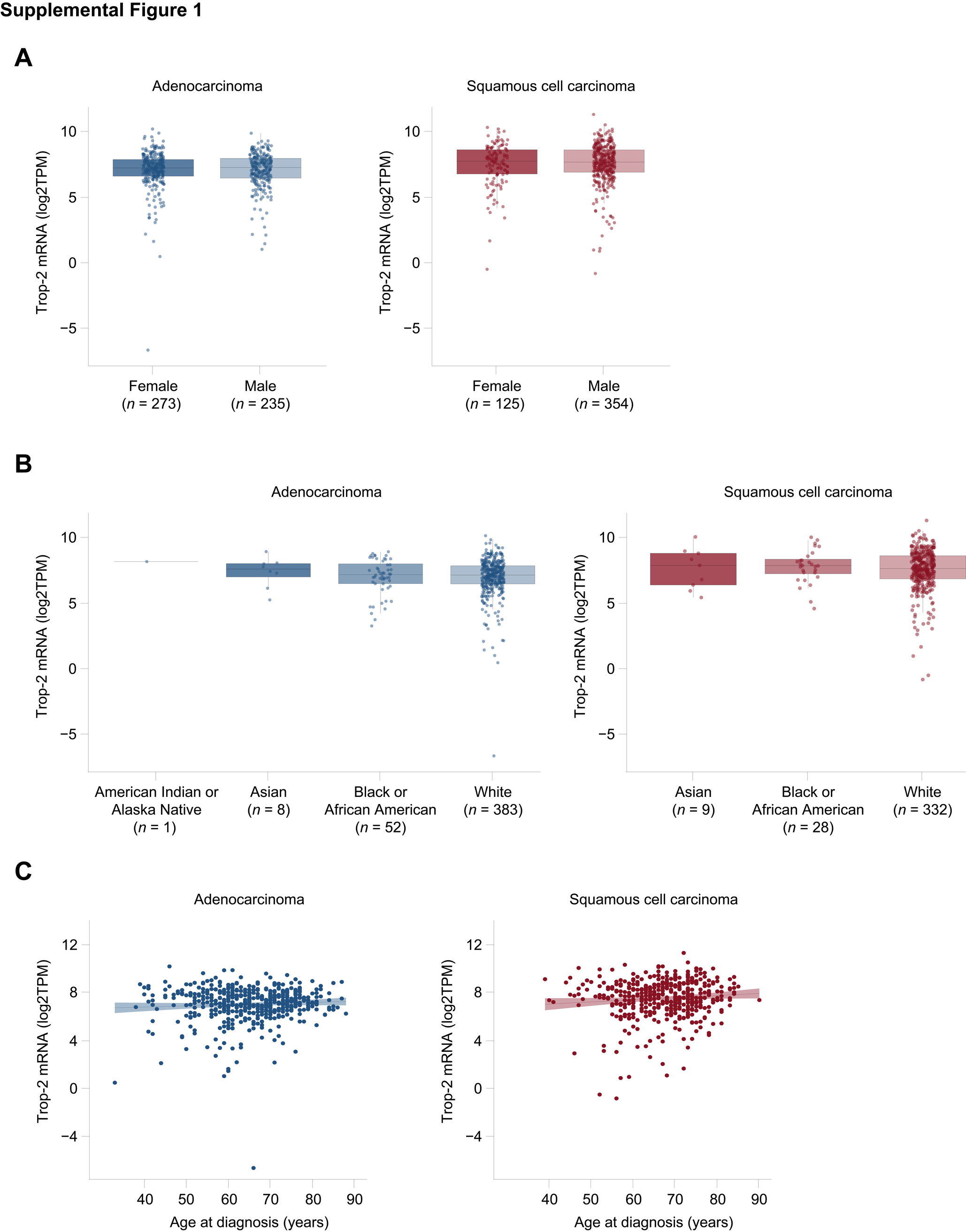

Supplement: S1 Fig — Trop-2 expression by histological subtype and across sex, race, and age is shown. Abbreviations: NSCLC, non–small cell lung cancer; TCGA, The Cancer Genome Atlas; TPM, transcript count per million; Trop-2, trophoblast cell surface antigen 2. (TIF) [file pone.0321555.s001.tif]

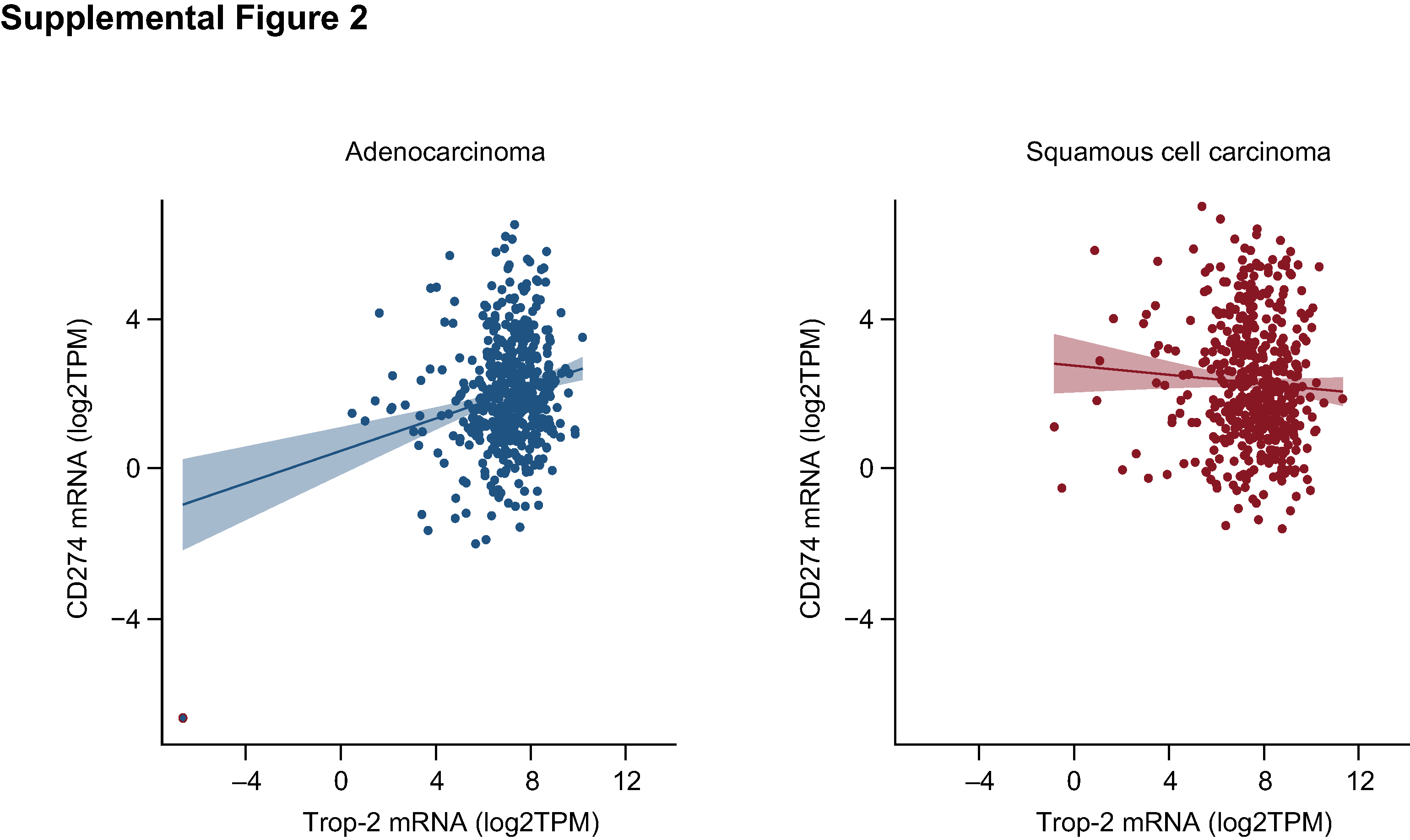

Supplement: S2 Fig — Abbreviations: CD, cluster of differentiation; LUAD, lung adenocarcinoma; LUSC, lung squamous cell carcinoma; PD-L1, programmed death-ligand 1. (TIF) [file pone.0321555.s002.tif]

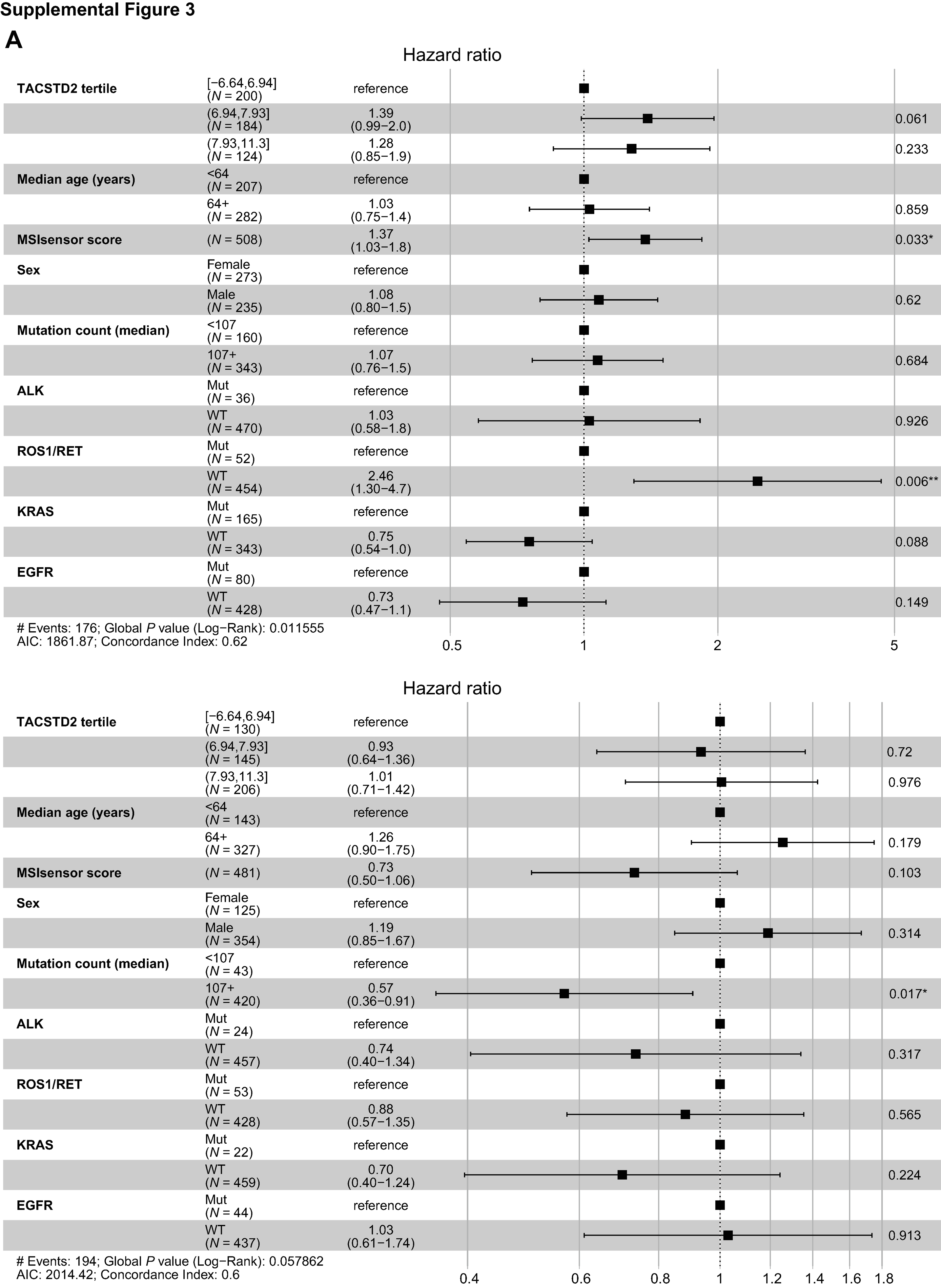

Supplement: S3 Fig — Abbreviations: ALK, anaplastic lymphoma kinase; EGFR, epidermal growth factor receptor; KRAS, Kirsten rat sarcoma viral oncogene homolog; Mut, mutant; RET, ret proto oncogene; ROS1, c ros oncogene 1; TACSTD2, tumor-associated calcium signal transducer 2; WT, wildtype. (TIF) [file pone.0321555.s003.tif]

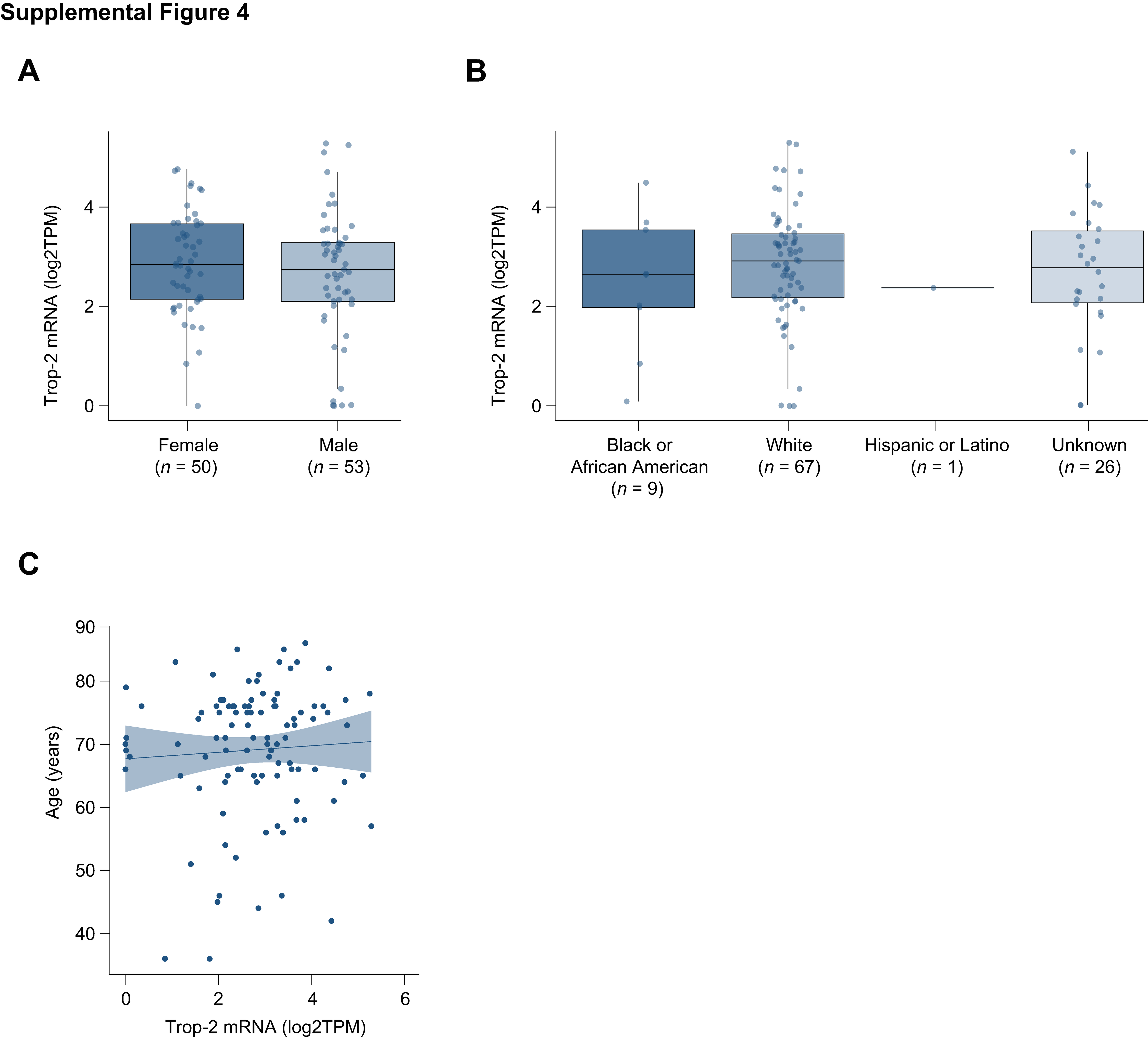

Supplement: S4 Fig — Trop-2 mRNA expression across sex (A), race (B) and correlation with age (C) are shown. (TIF) [file pone.0321555.s004.tif]

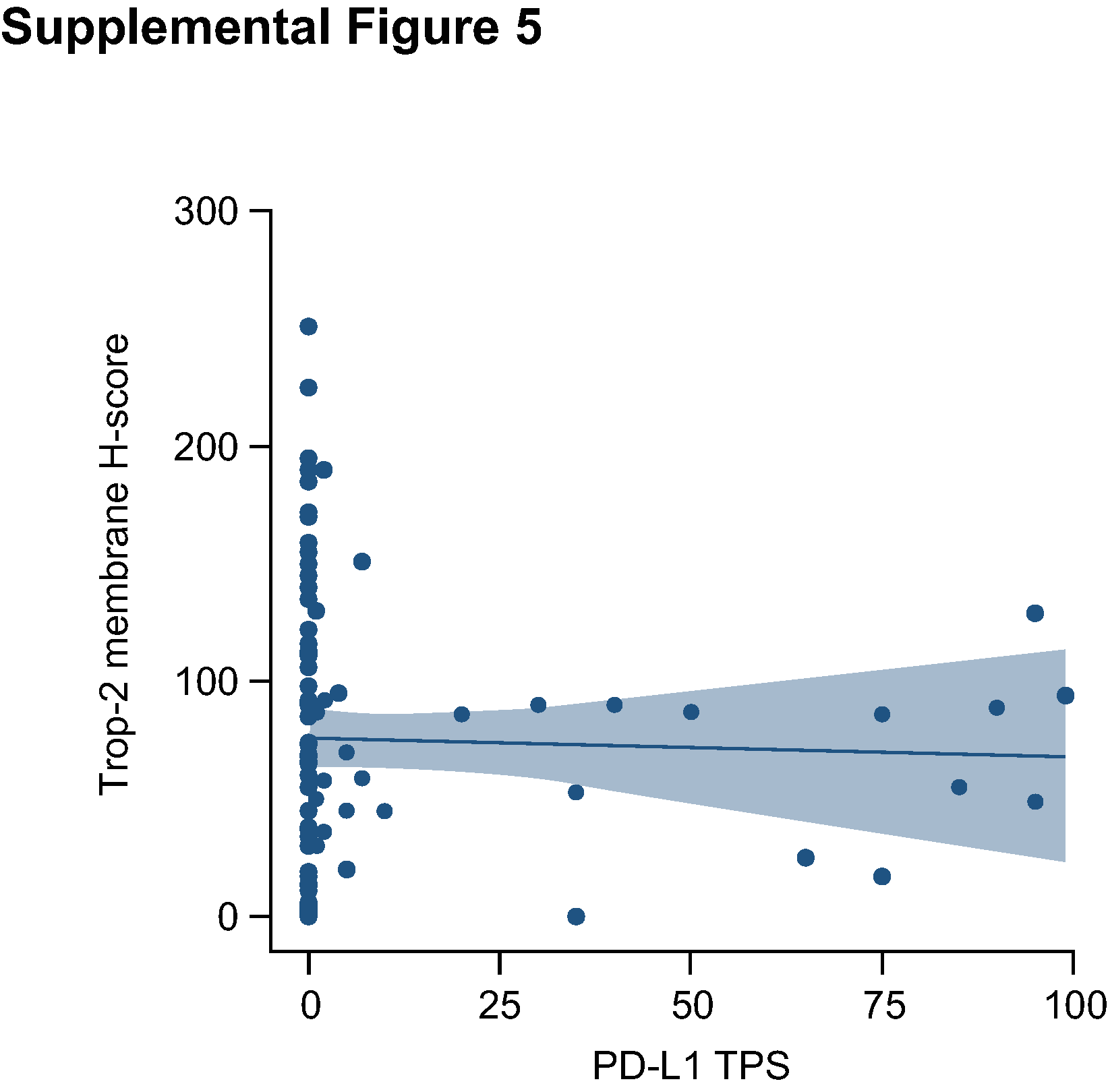

Supplement: S5 Fig — (TIF) [file pone.0321555.s005.tif]

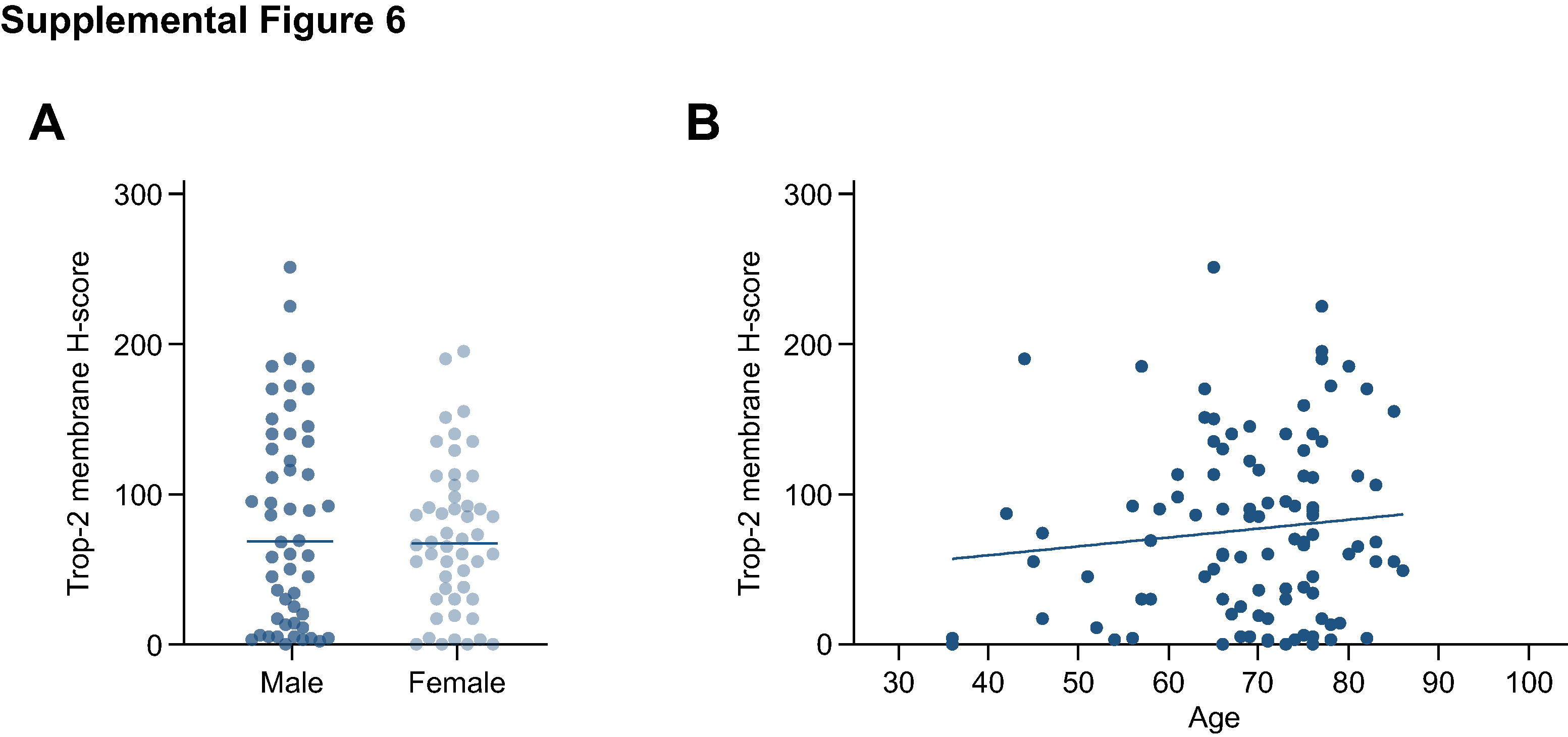

Supplement: S6 Fig — Trop-2 expression as H-score across sex (A) and correlation with age (B) is shown. (TIF) [file pone.0321555.s006.tif]

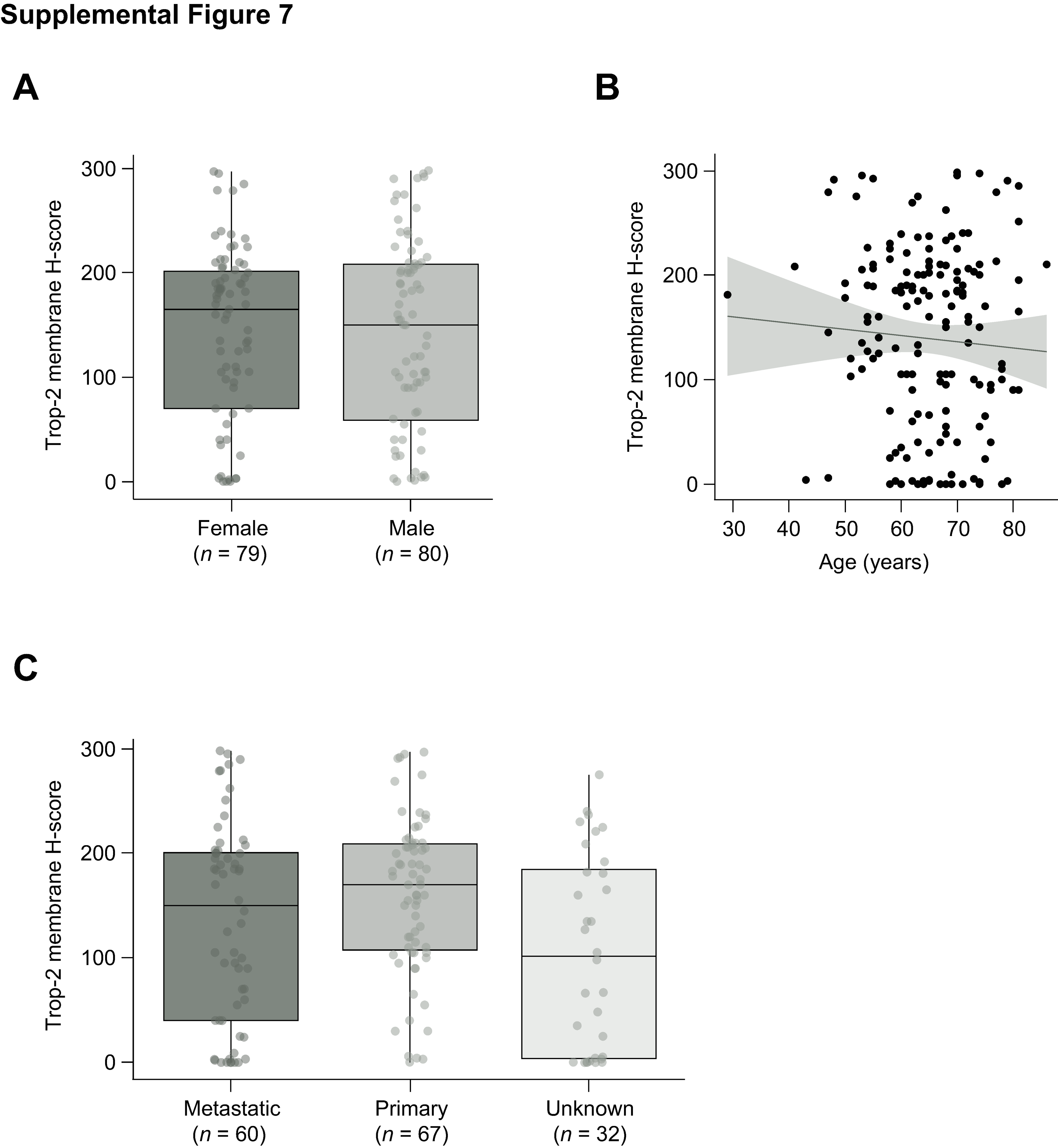

Supplement: S7 Fig — Trop-2 protein expression as H-score across sex (A), correlation with age (B), and between primary and metastatic tumors (C) is shown. (TIF) [file pone.0321555.s007.tif]

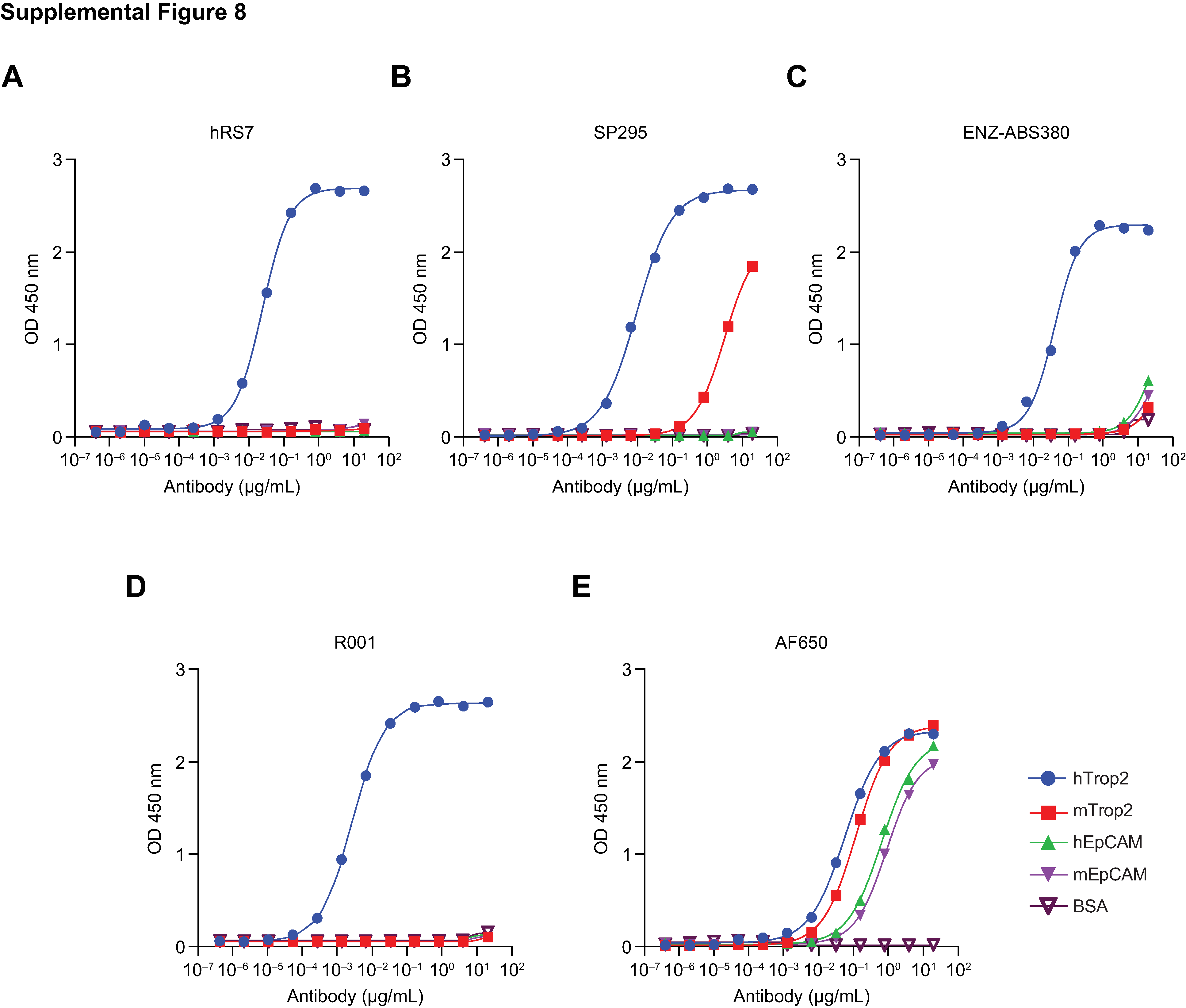

Supplement: S8 Fig — Humanized monoclonal antibody hRS7 IgG1ĸ of sacituzumab govitecan (A), rabbit monoclonal anti–Trop-2 (clone SP295) (B), mouse monoclonal anti–Trop-2 (clone ENZ-ABS380) (C), and rabbit monoclonal anti–Trop-2 (clone 001) specifically bind Trop-2 (D), and goat anti–Trop-2 (clone AF650) cross-reactivity to EPCAM/Trop-1 are shown (E). Abbreviations: BSA, bovine serum albumin; EPCAM, epithelial cell adhesion molecule; OD, optical density. (TIF) [file pone.0321555.s008.tif]
